# Education, collaboration, and innovation: intelligent biology and medicine in the era of big data

**DOI:** 10.1186/1471-2164-16-S7-S1

**Published:** 2015-06-11

**Authors:** Jianhua Ruan, Victor Jin, Yufei Huang, Hua Xu, Jeremy S Edwards, Yidong Chen, Zhongming Zhao

**Affiliations:** 1 Department of Computer Science, The University of Texas at San Antonio, 78249 San Antonio, TX, USA; 2 Department of Molecular Medicine, The University of Texas Health Science Center at San Antonio, 78229 San Antonio, TX, USA; 3 Department of Electrical and Computer Engineering, The University of Texas at San Antonio, 78249 San Antonio, TX, USA; 4 School of Biomedical Informatics, The University of Texas Health Science Center at Houston, 77030 San Antonio, TX, USA; 5 Department of Molecular Genetics and Microbiology, University of New Mexico, 87131 Albuquerque, NM, USA; 6 Greehey Children's Cancer Research Institute, The University of Texas Health Science Center at San Antonio, 78229 San Antonio, TX, USA; 7 Department of Epidemiology & Biostatistics, The University of Texas Health Science Center at San Antonio, 78229 San Antonio, TX, USA; 8 Department of Biomedical Informatics, Vanderbilt University School of Medicine, 37203 Nashville, TN, USA; 9 Department of Cancer Biology, Vanderbilt University School of Medicine, 37232 Nashville, TN, USA

## Abstract

Here we present a summary of the 2014 International Conference on Intelligent Biology and Medicine (ICIBM 2014) and the editorial report of the supplement to BMC Genomics and BMC Systems Biology that includes 20 research articles selected from ICIBM 2014. The conference was held on December 4-6, 2014 at San Antonio, Texas, USA, and included six scientific sessions, four tutorials, four keynote presentations, nine highlight talks, and a poster session that covered cutting-edge research in bioinformatics, systems biology, and computational medicine.

## Introduction

The 2014 International Conference on Intelligent Biology and Medicine (ICIBM 2014) was held on December 4-6, 2014 in downtown San Antonio, Texas, a popular city with the world famous River Walk filled with live music. More than one hundred researchers with diverse background spanning biology, medicine, computer science and engineering, statistics, and mathematics, among others attended the three-day event. The event promoted all attendees to exchange ideas, showcase recent innovative work, and foster interdisciplinary and multidisciplinary research collaborations, and provided education and training opportunities to students and junior investigators in bioinformatics, systems biology, intelligent computing, and computational medicine.

The scientific program included six scientific sessions, four tutorials, four keynote presentations, nine highlight talks, and a poster session. The details of all presentations are available on the conference website [1] and in the conference program book. Here we briefly review the keynote speakers' lectures, followed by the tutorials, and regular scientific sessions.

## Keynote lectures

Four keynote speakers who are world-renowned leaders in bioinformatics, genomics, systems biology and computational medicine delivered lectures on their cutting-edge research and shared their views and perspectives of their research fields. These speakers were Dr. Tim Huang from University of Texas Health Science Center at San Antonio, Dr. Josh Stuart from University of California, Santa Cruz, Dr. Lynda Chin from University of Texas MD Anderson Cancer Center, and Dr. Jasmine Zhou from University of Southern California.

*"Single-Cell Analysis of Tumor Heterogeneity" *Intratumor heterogeneity is a critical impediment to improving prognosis of many types of cancer such as breast and prostate cancer. In his lecture, Dr. Tim Huang presented a quantitative model to identify subpopulations of prostate single cells extracted from urine. Using a binary-coding system to identify unique concordant expression patterns among genes, the model resulted in a digital rendering of single-cell gene expression which enables non-invasive prognosis of prostate cancer patients. Dr. Tim Huang is Alice P. McDermott President's Distinguished Professor and Professor and Chair of the Department of Molecular Medicine at the University of Texas Health Science Center, San Antonio. He is also the holder of Max and Minnie Tomerlin Voelcker Distinguished University Professor and Deputy Director of the Cancer Therapy and Research Center, San Antonio. Dr. Huang is a Fellow of the American Association for the Advancement of Science.

*"Identifying All of Cancer's Manifestations through Integrated Pan Cancer Analysis" *Dr. Josh Stuart presented the recent work of The Pan-Cancer Initiative of The Cancer Genome Atlas (TCGA) Research Network, including the analysis of thousands of human tumors to discover molecular aberrations at the DNA, RNA, protein and epigenetic levels in order to uncover data-driven tumor subtypes. Dr. Stuart described an integrated picture of commonalities, differences and themes across tumor lineages emerging from this large-scale, comprehensive work. While cancers are primarily classified on the basis of the body location where the disease originates, according to their new study, however, one in ten cancer patients would be classified differently using a new classification system based on molecular subtypes instead of the current tissue-of-origin system. Analysis of the molecular aberrations and their functional roles across tumor types will teach us how to extend therapeutics effective in one cancer type to others with a matching genomic profile, and to construct personalized networks for use in developing combinatorial therapy. Dr. Josh Stuart is Professor of Biomolecular Engineering and Associate Director of Center Biomolecular Science and Engineering at the University of California, Santa Cruz. He co-leads a Genome Data Analysis Center for the TCGA project, co-chairs the pan-cancer TCGA effort, is a leader of the bioinformatics pathways group for the International Cancer Genome Consortium (ICGC), and directs the computational pathway analysis for a Stand Up To Cancer (SU2C) Dream Team to identify therapies for resistant prostate cancer. He is an Alfred P. Sloan Fellow, and received an NSF CAREER award in 2009.

*"Genomic Medicine: Transforming Cancer Research and Care" *Dr. Lynda Chin described a new model of integration, collaboration and cooperation between the research and clinical care enterprises, and between academia and industry, to bring to bear the power of technology and patient data on the cancer problem. She discussed the MD Anderson Cancer Center's APOLLO-Big Data platform and a prototype consumer-centric Amazon-like care delivery ecosystem in partnership with major industry giants, including IBM-Watson, to build MD Anderson Oncology Expert Advisor™cognitive decision support system. Dr. Lynda Chin is Professor and Chair in the Department of Genomic Medicine, The University of Texas MD Anderson Cancer Center. For the 13 years prior to joining the University of Texas MD Anderson Cancer Center in 2011, Dr. Chin had been a professor of dermatology at Dana-Farber Cancer Institute and Harvard Medical School and a senior associate member of the Broad Institute of MIT and Harvard, and the Scientific Director of the Belfer Institute for Applied Cancer Science. Dr. Chin is the founding chair of the Department of Genomic Medicine at MD Anderson Cancer Center. She also serves as the scientific director of the Institute for Applied Cancer Science, which merges the best attributes of academia and industry to enable science-driven drug discovery. Dr. Chin was elected a member of the Institute of Medicine of the National Academies in 2012.

*"Make Big Data Useful: Horizontal and Vertical Data Integration to Study Genes, Networks and Diseases" *Dr. Jasmine Zhou presented several interesting machine learning and graph algorithms for integrating data of the same type (e.g. gene expression), and of different types of data, such as epigenetic data, gene expression, and genome structures, in order to answer novel biological questions. She presented her recent work in integrating many RNA-seq datasets to perform high-resolution functional annotation of human genome, namely, predicting the functions of individual transcript isoforms, transforming the public gene expression repositories into a disease diagnosis database, and identifying multi-layer coordinated perturbation on cancer pathways from TCGA data. She also talked about a recent work on integrating the 3D chromatin structures, epigenetic modification, and transcription factors to study gene regulation. Dr. Jasmine Zhou is a professor of biological sciences and computer science at the University of Southern California. Dr. Zhou is the PI of the NIH center for knowledge base on disease connections within the MAP Gen consortium. She was a recipient of several awards including an Alfred Sloan fellowship and a NSF Career award.

## Tutorials

ICIBM 2014 included four tutorial sessions that covered frontier research topics such as proteomics, metabolomics, metagenomics, single cell analysis, and next-generation sequencing and data analysis. These tutorials provided a wealth of information on these cutting-edge techniques and were well appreciated by the conference participants.

*"Next-generation Sequencing and Data Analysis" *This tutorial was given by Dr. Yunlong Liu from Indiana University -Purdue University Indianapolis and Dr. Kun Huang from The Ohio State University. The tutorial introduced various aspects of RNA-seq experimental design and data analysis, including experimental consideration, transcriptome alignment, gene expression analysis, and alternative splicing analysis. Dr. Huang also demonstrated how to use popular analysis tools in the Galaxy environment.

*"Proteomics and Metabolomics" *This tutorial was organized by Dr. Susan E Weintraub from The University of Texas Health Science Center at San Antonio, Dr. Steve Patrie from The University of Texas Southwestern Medical Center, and Drs. William R. Alley and Jianqiu (Michelle) Zhang from The University of Texas at San Antonio. This tutorial first provided a general introduction to mass spectrometry in proteomics research, followed by various topics that included top-down proteomics (proteomic evaluation of intact proteins), database searching of tandem mass-spectral data for glycopeptides, and large-scale comparison of protein expression over multiple samples in LC-MS/MS. The tutorial provided a useful overview of proteome informatics for biologists, bioinformaticians, computational researchers, and alike.

*"Single Cell Data Analysis" *Single cell analysis has emerged as a new paradigm for the study of biological and pathological heterogeneity. The innovative nanotechnology has made omics research possible at the single cell level, which sheds new light on the biological and pathological mechanisms and shows promise for clinical applications. This tutorial was organized by Drs. Chun-Liang Chen, Zhao Lai and Yidong Chen from The University of Texas Health Science Center at San Antonio and Dr. Chenghang Zong from Baylor College of Medicine. The tutorial introduced major elements of the single-cell analysis pipeline, as well as the technical challenges and methodology development of single cell whole genome and transcriptome sequencing.

*"Metagenomics" *This tutorial was given by Dr. Chittibabu (Babu) Guda, from the University of Nebraska Medical Center and Dr. Qunfeng Dong from the University of North Texas. They provided an introduction to metagomics and demonstrated the utility of popular tools used in the analysis of metagenomic data. In addition, Dr. Guda presented MetaID, an alignment-free n-gram based method that can accurately identify microorganisms at the strain level and estimate the abundance of each microorganism in a sample. Dr. Dong also described his own research efforts on developing phylogenetic-based machine-learning method using 16S rDNA sequence data for classification, and provided step-by-step tutorials on three publicly available metagenomics software tools, Mothur, METAGENassist, and Picrust.

## Scientific sessions and BMC Genomics / Systems Biology supplement issues

ICIBM 2014 had six regular scientific sessions where scientists presented their recent innovative research in the areas of bioinformatics, systems biology, intelligent computing, and computational medicine. The presentations were selected through a rigorous review process handled by a program committee of more than 70 experts in the field (see Conference Organization) based on their scientific merit and technical quality. These sessions were:

**Session I: **Gene Regulation and Protein Interaction Networks, I

**Session II: **Gene Regulation and Protein Interaction Networks, II

**Session III: **Algorithms in NGS Data Analysis

**Session IV: **Metagenomics and Bioinformatics Methods

**Session V: **Epigenetics and Systems Biology

**Session VI: **Biomarker Identification Methods

The details of each session, including session chairs, speakers, and the title and abstract of each talk are available on the conference website [1] and in the conference program book. Here, we provide an editorial report of the supplement to BMC Genomics / Systems Biology that includes 20 research papers selected from more than 60 submissions. (Two of the 20 articles are selected to be published in BMC Systems Biology while the others are in BMC Genomics. Decisions were made by the executive editor of the journals based on the journals' scope.) Each manuscript was reviewed by at least three reviewers and was substantially revised according to reviewers' critiques before acceptance into the supplement. These papers cover a diverse of topics in bioinformatics and computational systems biology. We grouped them into five categories as below.

### Systems approach for cancer biomarker discovery

Identifying biomarkers to classify cancer subtypes or to predict cancer outcomes is one of the most challenging problems in cancer biology. This supplement includes impressive progress in both the development of better statistical/computational methods and also the utilization of novel molecule types as biomarkers. Ow and Kuznetsov [2] described a novel statistical test to identify stable and novel biomarkers across multiple cohorts. In [3], Cui et al. performed a comprehensive analysis using RNA-seq data and discovered co-expressed modules of long non-coding RNAs (lncRNAs) as potentia biomarkers for prostate cancer. Using a network-based approach, a genome-wide analysis performed by Xu et al. [4] revealed strong links between colorectal cancer (CRC) and trimethylamine N-oxide (TMAO), which is a gut microbial metabolite of dietary meat and fat, underscoring opportunities for the development of new gut microbiome-dependent diagnostic tests and therapeutics for CRC. The study by Wang et al. [5] investigated the expression of transcriptional and post-transcriptional regulators and cancer-related genes across 9 cancer types, and found that expression of RNA-binding proteins (RBPs) was significantly changed across most studied cancer types, and their direct interaction partners are enriched by cancer-related genes, suggesting the cascade regulation effect of RBPs in carcinogenesis.

### Network and complex diseases

A challenge in studying the genetic causes of complex diseases such as cancer and diabetes is that such diseases are usually caused by combinatorial effects of many genes, gene products, and small molecules that interact with each other to form a complex interaction network. Continuing previous years' trend [6,7], several papers in this supplement demonstrated that integrative analysis that combines multiple data sources and utilizes interaction networks can often provide more stable and accurate results than analysis with a single data source or considering each gene as an independent entity. Wang et al. [8] described a novel computational method to discover potential drug sensitivity relevant cancer subtypes and identify driver mutation modules of individual subtypes by coupling differentially expressed genes based subtyping analysis with driver mutation network analysis. The evaluation results on two major types of cancer (breast and lung) revealed subtypes with significant survival time difference and distinct driver mutations of individual subtypes. The research findings can be used to help guide the repurposing of known drugs and their combinations in order to target these dysfunctional modules and their downstream signaling effectively for achieving personalized or precision medicine treatment. Using protein-protein interaction network to capture the functional relationships among genes, Zhong et al. [9] showed that for most cancer types, using somatic mutations in a small panel of genes to classify tumors into subtypes is more effective than using whole exome-based mutation analysis, signifying the power of network-based approaches in cancer subtyping. Zhu et al. [10] performed a comprehensive analysis of the somatic mutation and network characteristics of tumor suppressor genes (TSGs) and oncogenes (OCGs) based on the mutation data from the Pan-Cancer project. They found that TSGs usually have a higher mutation frequency than OCGs, while TSGs, OCGs, and drug targets tend to interact with each other on the human protein-protein interaction network. The results provided novel insight into the roles of TSGs and OCGs in cancer development and treatment. Shi et al. [11] proposed a novel computational method to identify protein-protein interaction subnetworks that contain genes relevant to progression and recurrence of breast cancer; survival analysis based on the identified subnetworks improved the results of classifying the recurrence status of breast cancer patients.

Malaria is one of the major causes of mortality around the world and the effectiveness of antimalarial drugs has been constantly challenged during the past decades due to the fast evolution of parasites that are resistant to multiple lines of drugs. Chen and Xu [12] developed a random walk-based approach to integrate human-human, parasite-parasite, and human-parasite protein-protein interaction network to predict malaria-associated genes. The method was subsequently validated by using known malaria-associated genes as well as novel malaria genes with literature supporting evidence. In another study [13], Cai et al. developed a subnetwork alignment algorithm to identify network components that may be involved in malaria pathogenesis, which created a new list of potential rational targets for antimalarial intervention.

### NGS data analysis and gene regulatory networks

Identification of cis-regulatory regions such as promoters, enhancers and transcription factor binding sites are fundamental tasks in deciphering the complex gene regulatory network. Several papers included in this supplement demonstrated that these elements can be more accurately predicted by integrating heterogeneous NGS data types such as DNA methylation data, histone modification marks and DNase I hypersensitivity sites, and by incorporating more accurate biophysical models of the protein-DNA interaction. For example, Hwang et al. [14] discovered several interesting DNA methylation-associated features that can improve the prediction of promoter regions, including the long-range low methylation around the regulatory regions, strong autocorrelation of the methylation levels, greater dynamic range of methylation levels, and overrepresented sequence motifs. In [15], Wang and colleagues proposed a new biophysical model of protein-DNA interactions, BayesPI2+, to model protein-DNA interaction. Their method distinguishes itself from the existing approaches in that both strong and weak interactions are considered, which allowed their method to utilize the full extent of in vivo protein-DNA interaction data rather than only the top few hundred or thousand strongest binding sites. The implemented computer program is publicly available at [16]. Lastly, Zhang et al. [17] proposed a novel de novo motif finding algorithm, MOST+, which integrates genomic sequences and genome-wide signals such as intensity and shape features from histone modification marks and DNase I hypersensitivity sites, to improve the prediction accuracy. MOST+ can detect motifs from a large input sequence of about 100Mbs within a few minutes.

### Systems pharmacology and drug discovery

Using RNA-seq, Chen et al. [18] performed transcriptomic analysis on several different tissues of *Astragalus mongolicus*, one of the most important herbs used in traditional Chinese medicine, and revealed a comprehensive profile of metabolic activities among tissues for the production of bioactive compounds. The work provided valuable resources for bioengineering and in vitro synthesis of the natural compounds for medical research and for potential drug development. One major challenge in personalized medicine research is to identify the environmental factors that can alter drug response, and to investigate their molecular mechanisms. Combining bioinformatic analysis and literature mining, Philips et al. [19] discovered a potential interaction between Vitamin A and the aromatase gene (CYP19A1), a target for treatment of various cancers. This interaction was validated in three different cell lines (JEG3, HeLa, and LNCaP), thus proving its potential to be utilized in therapeutic treatment decision. Network-assisted computational pharmacology is a promising method for drug repurposing inference. In [20], Huang et al. created a weighted and integrated drug-target interactome (WinDTome) by combining drug-target interactions from six commonly used data sources, and used it to predict drugs for schizophrenia (SCZ). Starting from 41 known SCZ drugs and their targets, they inferred a total of 264 drugs that might have potential for SCZ treatment, among which 39 have been investigated in clinical trials for SCZ treatment and 74 for the treatment of other mental disorders, respectively, demonstrating the effectiveness of their approach.

### Intelligent computing

Efficient and effective computational methods are essential for solving many computational biology and computational medicine challenges. This supplement included several papers with novel computational methods, some of which are already described above in the other categories. Here we briefly describe the remaining papers in this category. In [21], Chen et. al proposed an algorithm, XBSeq, to analyze RNA-seq data. In contrast to most existing methods, their algorithm explicitly takes into consideration non-exonic mapped reads. According to their results, it can provide more accurate expression measurement and detect differential expressed genes even in noisy conditions. In [22], Mohammed and Guda presented an ensemble machine-learning method called ECemble to identify enzymes and enzyme classes from metagnomics data. Using ECemble on the human gut microbiome, they identified 48 canonical human metabolic pathways that have at least one bacteria-encoded enzyme, which demonstrated the complementary role of gut microbiome in human gut metabolism. In another example, Nguyen et al. [23] proposed a novel method to integrate the results of pathway enrichment analysis from multiple, partially overlap, and sometimes inconsistent data sources. By representing the pathways as Boolean functions, they were able to condense the overall results into a functional pathway-process network. Applying the method to analyze genes involved in myocardial infarction (MI), they found multiple biological processes with immediate impacts on MI responses. Lastly, Chiu et al. [24] developed a novel multiple regression-based approach, Covariability-based Multiple Regression (CoMRe), to construct gene regulatory networks in breast cancer. By using this approach, they identified ESR1 and ERBB2 as co-modulators related to hormone stimulus and tumorigenesis, which was subsequently validated in two independent datasets.

## Conference organization

### 2014 International Conference on Intelligent Biology and Medicine (ICIBM 2014)

(December 4-6, 2016, San Antonio, Texas, USA)

Our sincerest thanks to the members of our Steering, Program, Publication, Workshop/Tutorial, Award, Publicity, Trainee, and Local Organization committees, as well as our numerous reviewers and volunteers, for their countless hours and energy spent to make ICIBM 2014 a success! We could not have accomplished so much without the dedication of each and every person that contributed to this conference.

**Sponsors **National Science Foundation, University of Texas at San Antonio, University of Texas Health Science Center at San Antonio, Vanderbilt University (VU), Vanderbilt Center for Quantitative Sciences, Bioinformatics Resource Center at Vanderbilt-Ingram Cancer Center.

**Partners **Trinity University, Texas State University at San Marcos, Indiana University -Purdue University Indianapolis, International Society of Intelligent Biological Medicine, Shanghai Center for Bioinformation Technology, China, Shanghai Institute for BioMedicine, China, and Shanghai Jiao Tong University, China.

**General Chairs **Zhongming Zhao (Vanderbilt University) and Victor Jin (University of Texas Health Science Center at San Antonio).

**Steering Committee **Yidong Chen (University of Texas Health Science Center at San Antonio), Ramana Davuluri (Northwestern University), Jason Ernst (University of California, Los Angeles), Laura Elnitski (National Human Genome Research Institute), Paul Horton (Computational Biology Research Center, AIST, Japan), Tony Hu (Drexel University), Victor Jin (University of Texas Health Science Center at San Antonio), Wei Li (Baylor College of Medicine), Shili Lin (Ohio State University), Zhongming Zhao (Vanderbilt University)

**Program Committee **Chair: Yidong Chen (University of Texas Health Science Center at San Antonio), Co-Chair: Jeremy Edwards (University of New Mexico Health Sciences Center), Members: Kristen Anton (Dartmouth College), Yong-sheng Bai (Indiana State University), William S. Bush (Vanderbilt University), Jake Chen (Indiana University -Purdue University Indianapolis), Xue-Wen Chen (Wayne State University), Juan Cui (University of Georgia), Qinghua Cui (Peking University, China), Youping Deng (Rush University Medical Center), Joshua Denny (Vanderbilt University), Jennifer M. Fettweis (Virginia Commonwealth University), Marcelo Fiszman (National Library of Medicine, National Institutes of Health), Jan Freudenberg (Feinstein Medical Research Institute), Ge Gao (Peking University, China), Chittibabu (Babu) Guda (University of Nebraska Medical Center), Yan Guo (Vanderbilt University), Steve Horvath (University of California, Los Angeles), Tzu-Hung Hsiao (University of Texas Health Science Center at San Antonio), Weichun Huang, (National Institute of Environmental Health Sciences), Yang Huang (Kaiser Permanente), Yufei Huang (University of Texas at San Antonio), Jenn-Kang Hwang (National Chiao Tung University, Taiwan), Peilin Jia (Vanderbilt University), Yufang Jin (University of Texas at San Antonio), Victor Jin (University of Texas Health Science Center at San Antonio), Sun Kim (Seoul National University, Korea), Judith Klein-Seetharaman (University of Warwick, UK), Dmitry Korkin (University of Missouri - Columbia), K.B. Kulasekera (University of Louisville), Jun Kong (Emory University), Fuhai Li (Houston Methodist hospital Research Institute), Leping Li (National Institute of Environmental Health Sciences), Liao Li (University of Delaware), Honghuang Lin (Boston University), Chunyu Liu (University of Chicago), Hongfang Liu (Georgetown University), Qi Liu (Vanderbilt University), Tianming Liu (University of Georgia), Yin Liu (University of Texas Health Science Center at Houston), Yunlong Liu (Indiana University -Purdue University Indianapolis), Zhandong Liu (Baylor College of Medicine), Xinghua Lu (University of Pittsburgh), Zhiyong Lu (NCBI, National Library of Medicine), Patricio A. Manque (Universidad Mayor, Chile), Tabrez Mohammad (University of Texas Health Science Center at San Antonio), Ranadip Pal (Texas Tech University), Yonghong Peng (University of Bradford, United Kindom), Horacio Perez-Sanchez (Catholic University of Murcia, Spain), Jiang Qian (Johns Hopkins University), Thomas Rindflesch (National Institutes of Health), Marylyn Ritchie (Penn State University), Bairong Shen (Soochow University, China), Alexander Statnikov (New York University Langone Medical Center), Jingchun Sun (Vanderbilt University), Wing-Kin Sung (National University of Singapore, Singapore), P.S. Thiagarajan (National University of Singapore, Singapore), Manabu Torii (Georgetown University Medical Center), Jun Wan (Johns Hopkins University), Edwin Wang (National Council of Canada/McGill University), Jing Wang (Vanderbilt University), Junbai Wang (Oslo University Hospital, Norway), Yufeng Wang (University of Texas at San Antonio), Xiaoyan Wang (University of Connecticut), Chaochun Wei (Shanghai Jiao Tong University), Xiwei Wu (City of Hope National Medical Center), Yonghui Wu (University of Texas Health Science Center at Houston), Junfeng Xia (Anhui University, China), Yang Xiang (Ohio State University), Lu Xie (Shanghai Center for Bioinformation Technology, China), Hua Xu (The University of Texas Health Science Center at Houston), Jianhua Xuan (Virginia Tech), Zhenqing Ye (University of Texas Health Science Center at San Antonio), Sungroh Yoon (Seoul National University, Korea), Bing Zhang (Vanderbilt University), Michelle Zhang (University of Texas at San Antonio), Yanqing Zhang (Georgia State University), Min Zhao (University of the Sunshine Coast, Australia), Huiru (Jane) Zheng (University of Ulster, United Kingdom), W. Jim Zheng (University of Texas Health Science Center at Houston), and Dongxiao Zhu (Wayne State University).

**Publication Committee **Chair: Jianhua Ruan (University of Texas at San Antonio), Co-Chair: Lang Li (Indiana University -Purdue University Indianapolis).

**Workshop/Tutorial Committee **Chair: Yufang Jin (University of Texas at San Antonio), Co-Chair: Chittababu (Babu) Guda (University of Nebraska Medical Center).

**Award Committee **Chair: Yufeng Wang (University of Texas at San Antonio), Co-Chair: Hua Xu (The University of Texas Health Science Center at Houston).

**Publicity Committee **Chair: Dongxiao Zhu (Wayne State University), Co-Chair: Michelle Zhang (University of Texas at San Antonio).

**Trainee Committee **Chair: Mario Flores (University of Texas at San Antonio), Co-Chair: Qingguo Wang (Vanderbilt University).

**Local Organization Committee **Chair: Yufei Huang (University of Texas at San Antonio), Co-Chair: Tabrez Mohammad (University of Texas Health Science Center at San Antonio).

## Competing interests

The authors declare that they have no competing interests.

## Authors' contributions

JR, ZZ, YH, and VJ managed and participated in the peer-review of ICIBM'14 manuscripts, excluding those on which they were authors, and handled the editorial process of this supplement. YC, JE and HX supported the post-acceptance manuscript processing. JR, ZZ, YH and VJ wrote the article, which was read and approved by all authors. All authors have made substantial contribution to the collection of the manuscripts and abstracts and, thus, the success of the ICIBM 2014 conference.
